# The Performance of Emotion Classifiers for Children With Parent-Reported Autism: Quantitative Feasibility Study

**DOI:** 10.2196/13174

**Published:** 2020-04-01

**Authors:** Haik Kalantarian, Khaled Jedoui, Kaitlyn Dunlap, Jessey Schwartz, Peter Washington, Arman Husic, Qandeel Tariq, Michael Ning, Aaron Kline, Dennis Paul Wall

**Affiliations:** 1 Department of Pediatrics Stanford University Stanford, CA United States; 2 Department of Biomedical Data Science Stanford University Stanford, CA United States; 3 Department of Mathematics Stanford University Stanford, CA United States; 4 Department of Psychiatry and Behavioral Sciences Stanford University Stanford, CA United States

**Keywords:** mobile phone, emotion, autism, digital data, mobile app, mHealth, affect, machine learning, artificial intelligence, digital health

## Abstract

**Background:**

Autism spectrum disorder (ASD) is a developmental disorder characterized by deficits in social communication and interaction, and restricted and repetitive behaviors and interests. The incidence of ASD has increased in recent years; it is now estimated that approximately 1 in 40 children in the United States are affected. Due in part to increasing prevalence, access to treatment has become constrained. Hope lies in mobile solutions that provide therapy through artificial intelligence (AI) approaches, including facial and emotion detection AI models developed by mainstream cloud providers, available directly to consumers. However, these solutions may not be sufficiently trained for use in pediatric populations.

**Objective:**

Emotion classifiers available off-the-shelf to the general public through Microsoft, Amazon, Google, and Sighthound are well-suited to the pediatric population, and could be used for developing mobile therapies targeting aspects of social communication and interaction, perhaps accelerating innovation in this space. This study aimed to test these classifiers directly with image data from children with parent-reported ASD recruited through crowdsourcing.

**Methods:**

We used a mobile game called *Guess What?* that challenges a child to act out a series of prompts displayed on the screen of the smartphone held on the forehead of his or her care provider. The game is intended to be a fun and engaging way for the child and parent to interact socially, for example, the parent attempting to guess what emotion the child is acting out (eg, surprised, scared, or disgusted). During a 90-second game session, as many as 50 prompts are shown while the child acts, and the video records the actions and expressions of the child. Due in part to the fun nature of the game, it is a viable way to remotely engage pediatric populations, including the autism population through crowdsourcing. We recruited 21 children with ASD to play the game and gathered 2602 emotive frames following their game sessions. These data were used to evaluate the accuracy and performance of four state-of-the-art facial emotion classifiers to develop an understanding of the feasibility of these platforms for pediatric research.

**Results:**

All classifiers performed poorly for every evaluated emotion except happy. None of the classifiers correctly labeled over 60.18% (1566/2602) of the evaluated frames. Moreover, none of the classifiers correctly identified more than 11% (6/51) of the angry frames and 14% (10/69) of the disgust frames.

**Conclusions:**

The findings suggest that commercial emotion classifiers may be insufficiently trained for use in digital approaches to autism treatment and treatment tracking. Secure, privacy-preserving methods to increase labeled training data are needed to boost the models’ performance before they can be used in AI-enabled approaches to social therapy of the kind that is common in autism treatments.

## Introduction

### Background

Autism spectrum disorder (ASD) is a neurodevelopmental disorder characterized by stereotyped and repetitive behaviors and interests as well as deficits in social interaction and communication [1]. In addition, autistic children struggle with facial affect and may express themselves in ways that do not closely resemble those of their peers [2-4]. The incidence of ASD has increased in recent years; it is now estimated that approximately 1 in 40 children in the United States is affected by this condition [5]. Although autism has no cure, there is strong evidence that suggests early intervention can improve speech and communication skills [6].

Common approaches to autism therapy include applied behavior analysis (ABA) and the early start Denver model (ESDM). In ABA therapy, the intervention is customized by a trained behavioral analyst to specifically suit the learner’s skills and deficits [7]. The basis of this program is a series of structured activities that emphasize the development of transferable skills to the real world. Similarly, naturalistic developmental behavioral interventions such as ESDM support the development of core social skills through interactions with a licensed behavioral therapist while emphasizing joint activities and interpersonal exchange [8]. Both treatment types have been shown to be safe and effective, with their greatest impact potential occurring during early intervention at younger ages [9-11].

Despite significant progress in understanding this condition in recent years, imbalances in coverage and barriers to diagnosis and treatment remain. In developing countries, studies have noted a lack of trained health professionals, inconsistent treatments, and an unclear pathway from diagnosis to intervention [12-14]. Within the United States, research has shown that children in rural areas receive diagnoses approximately 5 months later than children living in cities [15]. Moreover, it has been observed that children from families near the poverty line receive diagnoses almost a full year later than those from higher-income families. Data-driven approaches have estimated that over 80% of US counties contain no diagnostic autism resources [16]. Even months of delayed access to therapy can limit the effectiveness of subsequent behavioral interventions [15]. Alternative solutions that can ameliorate some of these challenges could be derived from digital and mobile tools. For example, we developed a wearable system using Google Glass that leverages emotion classification algorithms to recognize the facial emotion of a child’s conversation partner for real-time feedback and social support and showed treatment efficacy in a randomized clinical trial [17-25].

Various cloud-based emotion classifiers may help the value and reach of mobile tools and solutions. These include four commercially available systems: Microsoft Azure Emotion application programming interface (API) [26], Amazon Rekognition [27], Google Cloud Vision [28], and Sighthound [29]. Whereas most implementations of these emotion recognition APIs are proprietary, these algorithms are typically trained using large facial emotion datasets such as the Cohn-Kanade database [30] and Belfast-Induced Natural Emotion Database [31], which have few examples of children. Due to this bias in labeled examples, it is possible that these models do not generalize well to the pediatric population, including children with developmental delays such as autism, which is evaluated in this study. This study puts the disparity to test. To do so, we use our mobile game *Guess What?* [32-35]. This game (native to Android [36] and iOS [37] platforms) fosters engagement between the child and their social partner, such as a parent, through charades-like games while building a database of facial image data enriched for a range of emotions exhibited by the child during the game sessions.

**Figure 1:**
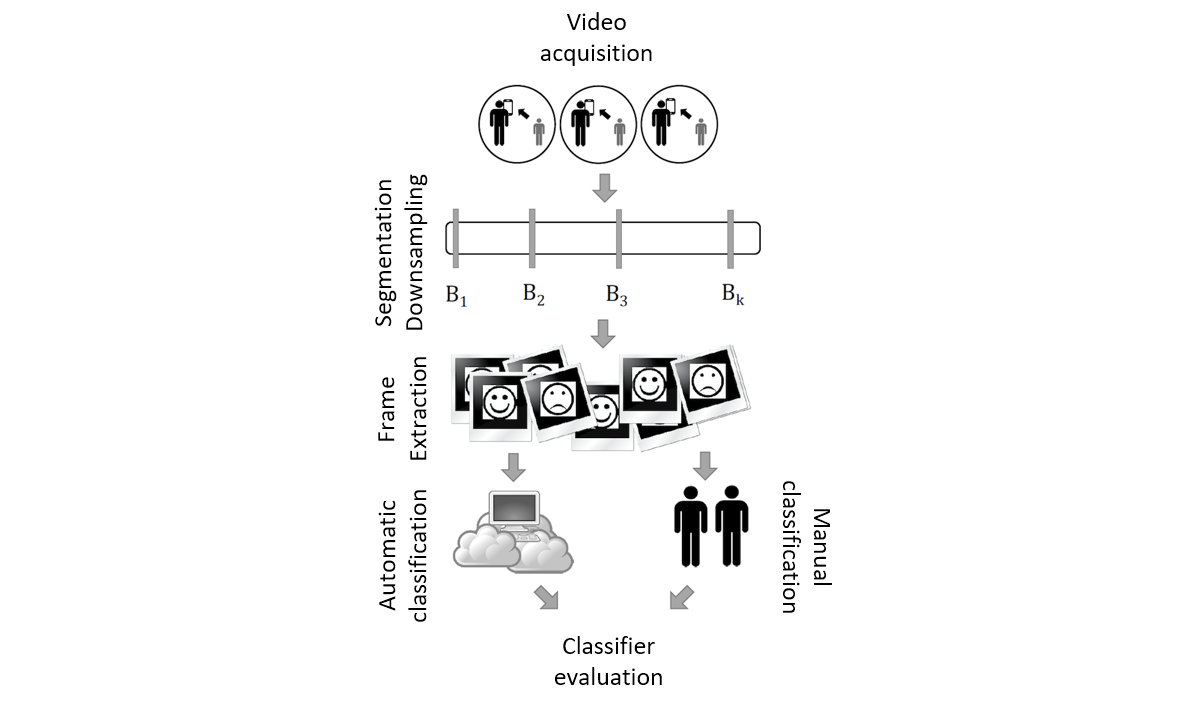
A mobile charades game played between caregiver and child is used to crowdsource emotive video, subsampled and categorized by both manual raters and automatic classifiers. Frames from these videos form the basis of our dataset to evaluate several emotion classifiers.

The primary contributions of this study are as follows:

1. We present a mobile charades game, Guess What?, to crowdsource emotive video from its players. This framework has utility both as a mechanism for the evaluation of existing emotion classifiers and for the development of novel systems that appropriately generalize to the population of interest.
2. We present a study in which 2602 emotive frames are derived from 21 children with a parent-reported diagnosis of autism using data from the Guess What mobile game collected in a variety of heterogeneous environments.
3. The data were used to evaluate the accuracy and performance of several state-of-the-art classifiers using the workflow shown in Figure 1, to develop an understanding of the feasibility of using these APIs in future mobile therapy approaches.

### Related Work

To the best of our knowledge, this is the first work to date that benchmarks public emotion recognition APIs on children with developmental delays. However, a number of interesting apps have been proposed in recent years, which employ vision-based tools or affective computing solutions as an aid for children with autism. The emergence of these approaches motivates a careful investigation of the feasibility of commercial emotion classification algorithms for the pediatric population.

Motivated by the fact that children with autism can experience cognitive or emotional overload, which may compromise their communication skills and learning experience, Picard et al [38] provided an overview of technological advances for sensing autonomic nervous system activation in real-time, including wearable electrodermal activity sensors. A more general overview of the role of affective computing in autism is provided by Kalioby et al [39], with the motivating examples of using technology to help individuals better navigate the socioemotional landscape of their daily lives. Among the enumerated devices include those developed at the Massachusetts Institute of Technology media laboratory, such as *expression glasses* that discriminate between several emotions, skin conductance-sensing gloves for stress detection, and a pressure-sensitive mouse to infer affective state from how individuals interact with the device. Devices made by industry include the SenseWear Pro2 armband, which includes a variety of wearable sensors that can be repurposed for stress and productivity detection, smart gloves that can detect breathing rate and blood pressure, and wireless heart-rate monitors that can be analyzed in the context of environmental stressors [40].

Prior research conducted by us has demonstrated the efficacy of mobile video phenotyping approaches for children with ASD in general [41-47] and via the use of emotion classifiers integrated with the Google Glass platform to provide real-time behavioral support to children with ASD [17-25]. In addition, other studies have confirmed the usability, acceptance, and overall positive impact on families of Google Glass–based systems that use emotion recognition technology to aid social-emotional communication and interaction for autistic children [48,49]. In addition to these efforts, a variety of other smart-glass devices have been proposed. For example, the SenseGlass [50] is among the earliest works that propose leveraging the Google Glass platform to capture and process real-time affective information using a variety of sensors. The authors proposed apps, including the development of affect-based user interfaces, and empowering wearers toward behavioral change through emotion management interventions.

Glass-based affect recognition that predates the Google Glass platform has also been proposed. Scheirer et al [51] used piezoelectric sensors to detect expressions such as confusion and interest, which were detected with an accuracy of 74%. A more recent work proposes a device called *Empathy Glasses* [52] in which users can see, hear, and feel from the perspective of another individual. The system consists of wearable hardware to transmit the wearers’ gaze and facial expression and a remote interface where visual feedback is provided, and data are viewed.

The research for smart-glass–based interventions is further supported by other technological systems that have been developed and examined within the context of developmental delays, including the use of augmented reality for object discrimination training [53], assistive robotics for therapy [54-56], and mobile assistive technologies for real-time social skill learning [57]. Furthermore, the use of computer vision and gamified systems to both detect and teach emotions continues to progress. A computational approach to detect facial expressions optimized for mobile platforms was proposed [58], which demonstrated an accuracy of 95% from a 6-class set of expressions. Leo et al [59] proposed a computational approach to assess the ability of children with ASD to produce facial expressions using computer vision, validated by three expert raters. Their findings demonstrated the feasibility of a human-in-the-loop computer vision system for analyzing facial data from children with ASD. Similar to this study, which utilizes *Guess What?*, a charades-style mobile game to collect emotional face data, Park and colleagues proposed six game design methods for the development of game-driven frameworks in teaching emotions to children with ASD, of which include: observation, understanding, mimicking, and generalization, and supports the use of game play to produce data of value to computer vision approaches for children with autism [60].

Although not all of the aforementioned research studies employ emotion recognition models directly, they are indicative of a general transition from traditional health care practices to modern mobile and digital solutions that leverage recent advances in computer vision, augmented reality, robotics, and artificial intelligence [61]. Thus, the trend motivates our investigation of the efficacy of state-of-the-art vision models on populations with developmental delay.

## Methods

### Overview

In this section, we describe the architecture of *Guess What?* followed by a description of the methods employed to obtain test data and processing the frames therein to evaluate the performance of several major emotion classifiers. Although dozens of APIs are available, we limit our analysis to some of the most popular systems from major providers of cloud services as a fair representation of the state-of-the-art in publicly available emotion recognition APIs. The systems evaluated in this work were Microsoft Azure Emotion API (Azure) [26], Amazon AWS Rekognition (AWS) [27], Google Cloud Vision API (Google) [28], and Sighthound (SH) [29].

### System Architecture

The evaluation of the state-of-the-art in public emotion classification APIs on children with ASD requires a dataset derived from subjects from the relevant population group with a fair amount of consistency in its format and structure. Moreover, as data are limited, it is critical that the video contains a high density of emotive frames to simplify the manual annotation process when establishing a ground truth. Therefore, we have developed and launched an educational mobile game on the Google Play Store [34] and iOS App Store [35], *Guess What?,* from which we derive emotive video.

In this game, parents hold the phone such that the front camera and screen are facing outward toward the child. When the game session begins, the child is shown a prompt that the caregiver must guess based on the child’s gestures and facial expressions. After a correct guess is acknowledged, the parent tilts the phone forward, indicating that a point should be awarded. At this time, another prompt is shown. If the one holding the phone cannot make a guess, he/she will tilt the phone backward to skip the frame and automatically proceed to the next. This process repeats until the 90-second game session has elapsed. Meta information is generated for each game session that indicates the times at which various prompts are shown and when the correct guesses occur.

**Figure 2:**
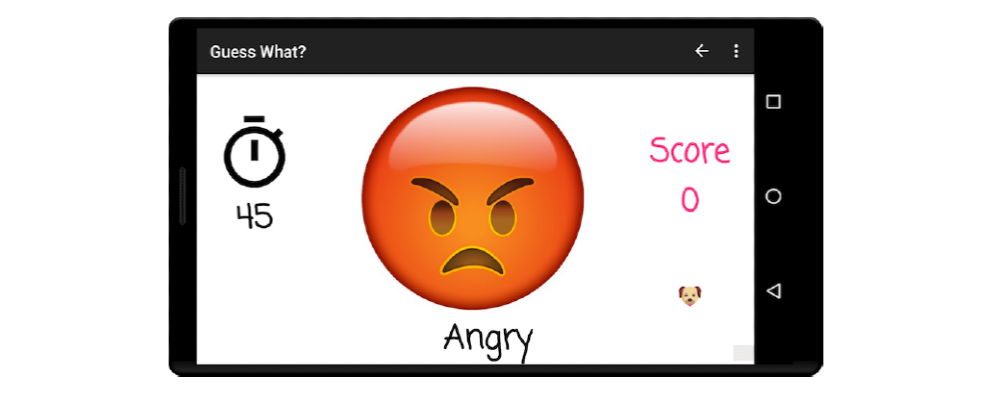
Prompts from the emoji category are caricatures, but many are still associated with the classic Ekman universal emotions.

**Figure 3:**
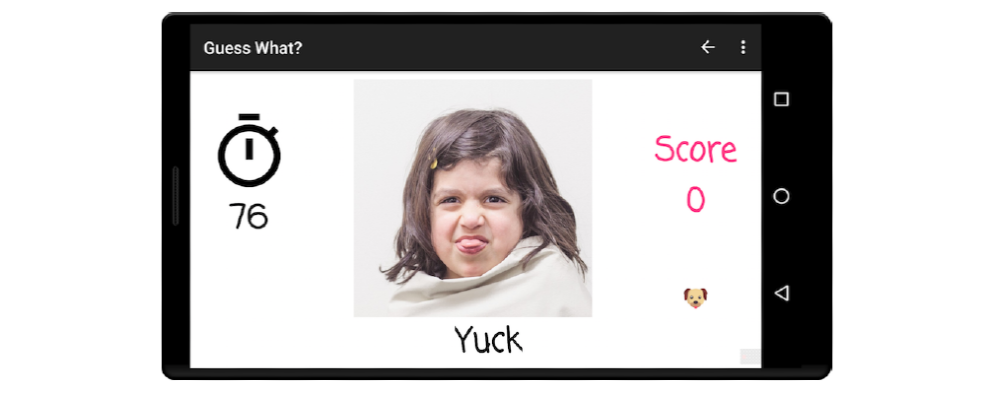
Prompts from the faces category are derived from real photos of children over a solid background.

Although a number of varied prompts are available, the two that are most germane to facial affect recognition and emotion recognition are emojis and faces, as shown in Figures 2 and 3, respectively. After the game session is complete, caregivers can elect to share their files and associated metadata to an institution review board-approved secure Amazon S3 bucket that is fully compliant with the Stanford University’s high-risk application security standards. A more detailed discussion of the mechanics and applications of *Guess What?* is described in [29-32].

**Figure 4:**
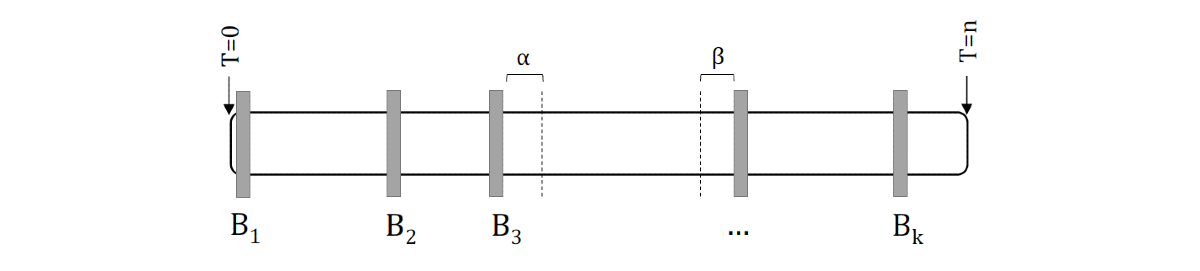
The structure of a single video is characterized by its boundary points, which identify the times at which various prompts were shown to the child.

The structure of a video is shown in Figure 4. Each uploaded video yields *n* video frames, delineated by *k* boundary points, *B_1_-B_k_*, where each boundary point represents the time at which a new prompt is shown to the user. To obtain frames associated with a particular emotion, one should first identify the boundary point associated with that emotion through the game meta information, *i*. Having identified this boundary point, frames between *B_i_* and *B_i_+1* can be associated with this prompt. However, two additional factors remain. It typically takes some time, *α*, for the child to react after the prompt is shown. Moreover, there is often a time period, *β*, between the child’s acknowledgment of the parents’ guess and phone tilt by the parent, during which time the child may adopt a neutral facial expression. Therefore, the frames of interest are those that lie between *B_i_+α* and *B_i+1_−β*.

The proposed system is centered on two key aims. First, this mechanism facilitates the acquisition of structured emotive videos from children in a manner that challenges their ability to express facial emotion. Whereas other forms of video capture could be employed, a gamified system encourages repeated use and has the potential to contain a much higher density of emotive frames than a typical home video structured around nongaming activities. As manual annotation is employed as a ground truth for evaluating emotion classification, a high concentration of emotive frames within a short time period is essential to the simplification and reduction of the burden associated with this process. A second aim is to potentially facilitate the aggregation of labeled emotive videos from children using a crowdsourcing mechanism. This can be used to augment existing datasets with labeled images or create new ones for the development of novel deep-learning-based emotion classifiers that can potentially overcome the limitations of existing methods.

### Data Acquisition

A total of 46 videos from 21 subjects were analyzed in this study. These data were collected over 1 year. Ten videos were collected in a laboratory environment from six subjects with ASD who played several games in a single session administered by a member of the research staff. An additional 36 videos were acquired through crowdsourcing from 15 remote participants. Diagnosis of any form of developmental disorder was provided by the caregiver through self-report during the registration process, along with demographic information (gender, age, ethnicity). The collected information included diagnoses of autistic disorder (autism), ASD, Asperger's syndrome, pervasive developmental disorder (not otherwise specified), childhood disintegrative disorder, no diagnosis, no diagnosis but suspicious, and social communication (pragmatic) disorder. Additionally, a free-text field was available for parents to specify additional conditions. The videos were evaluated by a clinical professional using the Diagnostic and Statistical Manual of Mental Disorders-V criteria before inclusion [1]. Caregivers of all children who participated in the study selected the *autism spectrum disorder* option.

The format of a *Guess What?* gameplay session generally enforces a structure on the derived video: the device is held in landscape mode, the child’s face is contained within the frame, and the distance between the child and camera is typically between 2 and 10 feet. Nevertheless, these videos were carefully screened by members of the research staff to ensure the reliability and quality of the data therein; videos that did not include children, were corrupt, filmed under poor lighting conditions, or did not include plausible demographic information were excluded from the analysis. The average age of the participating children was 7.3 (1.76) years. Due to the small sample size and nonuniform incidence of autism between genders [62], 18 of the 21 participants were male. Although participants explored a variety of game mechanics, all analyzed videos were derived from the two categories most useful for the study of facial affect: faces and emojis. After each game session, the videos were automatically uploaded to an Amazon S3 bucket through an Android background process.

### Data Processing

Most emotion classification APIs charge users per an http request, rendering the processing of every frame in a video prohibitive in terms of both time and cost. To simplify our evaluation, we subsampled each video at a rate of two frames per second. These frames formed the basis of our experiments. To obtain ground truth, two raters manually assigned an emotion label to each frame based on the seven Ekman universal emotions [63], with the addition of a neutral label. Some frames were discarded when there was no face, or the quality was too poor to make an assessment. A classifier’s performance on a frame was evaluated only under the conditions that the frame was valid (of sufficient quality), and the two manual raters agreed on the emotion label associated with the frame. Frames were considered of insufficient quality if: (1) the frame was too blurry to discern, (2) the child was not in the frame, (3) the image or video was corrupt, or (4) there were multiple individuals within the frame.

From a total of 5418 reviewed frames, 718 were discarded due to a lack of agreement between the manual raters. An additional 2123 frames were discarded because at least one rater assigned the *not applicable (N/A)* label, indicating that the frame was of insufficient quality. This was due to a variety of factors but generally caused by motion artifacts or the child leaving the frame due to the phone being tilted in acknowledgment of a correct guess. The total number of analyzed frames was 2602 divided between the categories shown in Table 1.

As shown, most frames were *neutral*, with a preponderance of *happy* frames in the nonneutral category. Owing to the limited number of *scared* and *confused* frames, this emotion was omitted from our analysis. We also merged the *contempt* and *anger* categories due to their similarity of affect and streamline analysis.

**Table 1:** The distribution of frames per category (N=2602).

Emotion	Frames, n
Neutral	1393
Emotive	1209
Happy	864
Sad	60
Surprised	165
Disgusted	69
Angry	51

As not all emotion classifiers represented their outputs in a consistent format, some further simplifications were made in our analysis. First, it was necessary to make minor corrections to the format of the outputted data. For example, *happy* and *happiness* were considered identical. In the case of AWS, the *confused* class was ignored, as many other classifiers did not support it. Moreover, *calm* was renamed *neutral*. As AWS, Azure, and Sighthound returned probabilities rather than a single label, a frame in which no emotion class was associated with a probability of over 70% was considered a failure. For Google Vision, classification confidence was associated with a categorical label rather than a percentage. In this case, frames did not receive an emotion classification as *likely* or *very likely* were considered failures. It is also worth noting that this platform, unlike all the others, does not contain *disgust* or *neutral* classes. The final emotions evaluated in this study were *happy*, *sad*, *surprise*, *anger*, *disgust*, and *neutral*, with the latter two omitted for Google Cloud Vision.

As real-time use is an important aspect of mobile therapies and aids, we evaluated the performance of each classifier by calculating the number of seconds required to process each 90-second video subsampled to one frame per second. This evaluation was performed on a Wi-Fi network tested with an average download speed of 51 Mbps and an average upload speed of 62.5 Mbps. For each classifier, this experiment was repeated 10 times to obtain the average amount of time required to process the subsampled video.

## Results

### Overview

In this section, we present the results of our evaluation of *Guess What?* as well as the performance of the evaluated classifiers: Microsoft Azure Emotion API (Azure) [26], AWS [27], Google Cloud Vision API (Google) [28], and SH [29]. Abbreviations for emotions described within this section can be found in Textbox 1.

**Abbreviations for emotions.**

HP: Happy

CF: Confused

N/A: Not applicable

SC: Scared

SP: Surprised

DG: Disgusted

AG: Angry

### Classifier Accuracy

#### Comparison With Ground Truth (Classifiers)

Table 2 shows the performance of each classifier calculated by the percentage of correctly identified frames compared with the ground truth for categories *neutral*, *emotive,* and *all*. A *neutral* frame is one in which the face is recognized, and the neutral label is assigned high confidence. Any other frame within the categories of *happy*, *sad*, *surprised*, *disgusted*, and *angry*, are considered emotive frames. A more detailed breakdown of performance by emotion is shown in Table 3. Note that as before, Google’s API does not support the *neutral* and *disgust* categories.

**Table 2:** Percentage of frames correctly identified by classifier: Azure (Azure Cognitive Services), AWS (Amazon Web Services), SH (Sighthound), and Google (Google Cloud Vision). These results only include frames in which there was a face, and the two manual raters agreed on the class. Google Vision API does not support the neutral label.

Classifier	Frame type		
	Emotive (n=1209), n (%)	Neutral (n=1393), n (%)	All (n=2602), n (%)
Azure	798 (66.00)	744 (53.40)	1542 (59.26)
AWS^a^	829 (68.56)	679 (48.74)	1508 (57.95)
Google	785 (64.92)	N/A^b^	N/A
Sighthound	664 (54.92)	902 (64.75)	1566 (60.18)

^a^AWS: Amazon AWS Rekognition.

^b^N/A: not applicable.

**Table 3:** Percentage of frames correctly identified by emotion type by each classifier: Azure (Azure Cognitive Services), AWS (Amazon Web Services), SH (Sighthound), and Google (Google Cloud Vision). These results only include frames in which there was a face, and the two manual raters agreed on the class. Note: Google Vision API does not support the neutral or disgust labels.

Classifier	Frame type					
	Neutral (n=1394), n (%)	Happy (n=864), n (%)	Sad (n=60), n (%)	Surprised (n=165), n (%)	Disgusted (n=69), n (%)	Angry (n=51), n (%)
AWS	679 (48.74)	709 (82.0)	19 (31)	94 (56.9)	4 (5)	3 (5)
Sighthound	902 (64.75)	545 (63.0)	13 (21)	90 (54.5)	10 (14)	6 (11)
Azure	744 (53.41)	695 (80.4)	20 (33)	80 (48.4)	0 (0)	3 (5)
Google	N/A^a^	676 (78.2)	10 (16)	93 (56.3)	N/A	6 (11)

^a^N/A: not applicable.

#### Interrater Reliability (Classifiers)

**Figure 5:**
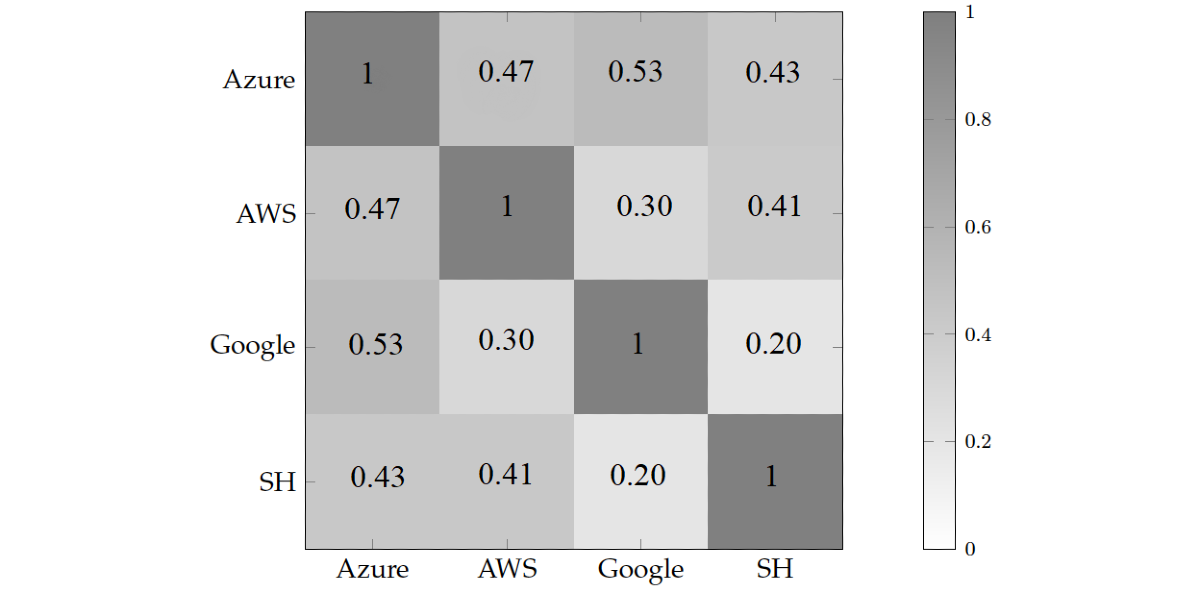
The Cohen’s Kappa Score is a measure of agreement between two raters, and was calculated for all four evaluated classifiers: Azure (Azure Cognitive Services), AWS (Amazon Web Services), SH (Sighthound), and Google (Google Cloud Vision). Results indicate weak agreement between all pairs of classifiers.

The Cohen kappa statistic is a measure of interrater reliability that factors in the percentage of agreement due to chance; an important consideration when the possible classes are few in number. Figure 5 shows the agreement between every pair of classifiers based on their Cohen kappa score calculated based on every evaluated frame, in which a score of 1 indicates perfect agreement. The results reflect low agreement between most combinations of classifiers. This is particularly true for the lack of agreement between Google and Sighthound, with a Cohen kappa score of 0.2. This is likely because of differences in how the classifiers are tuned for precision and recall; Sighthound correctly identified more *neutral* frames than the others, but performance was lower for the most predominant emotive label: *happy*.

#### Interrater Reliability (Human Raters)

**Figure 6:**
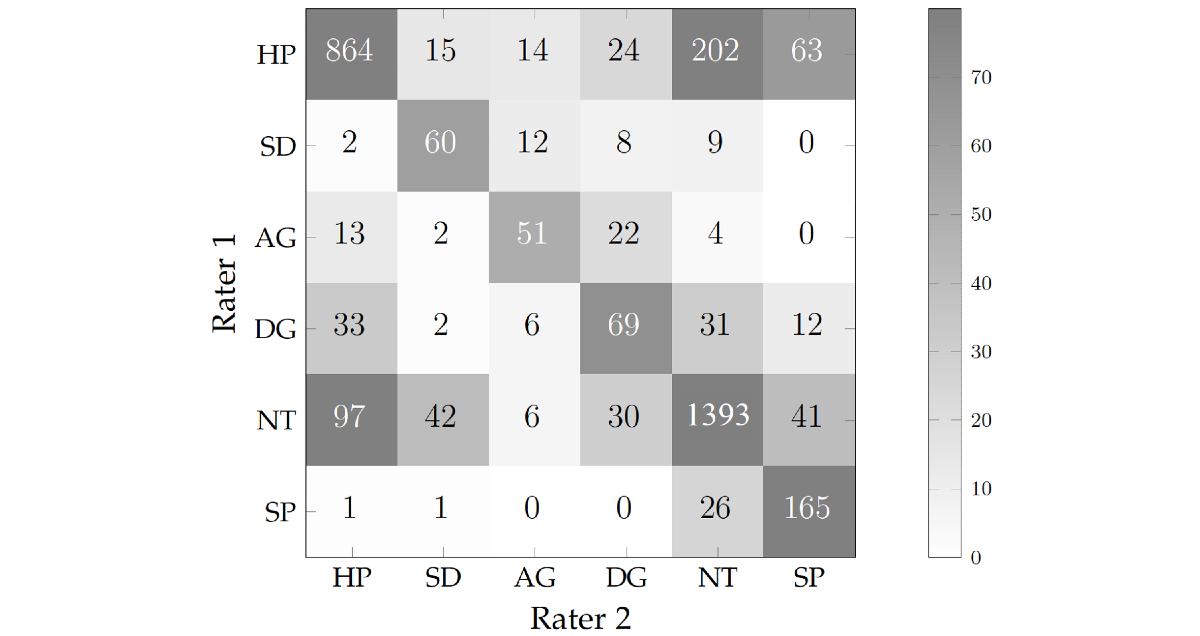
The distribution of frames between the two human raters for each emotion: HP (Happy), SD (Sad), AG (Angry), DG (Disgust), NT (Neutral), and SC (Scared).

The Cohen kappa coefficient for agreement between the two manual raters was 0.74, which was higher than any combination of automatic classifiers evaluated in this study. Although this indicates substantial agreement, it is worth exploring the characteristics of frames in which there was disagreement between the two raters. The full confusion matrix can be seen in Figure 6, which shows the distribution of all frames evaluated by the raters. The results indicate that most discrepancies were between happy and neutral. These discrepancies were likely subtle differences in how the raters perceived a happy face due to the inherent subjectivity of this process. A lack of agreement can also be seen between the disgust-anger categories.

### Classifier Speed

Wearable and mobile solutions for autism generally require efficient classification performance to provide real-time feedback to users. In some cases, this may be environmental feedback, as in the Autism Glass [17-25], which uses the outward-facing camera of Google Glass to read the emotions of those around the child and provide real-time social queues. In the case of *Guess What?* the phone’s front camera is used to read the expression of the child, which can be analyzed to determine if the facial expression matches the prompt displayed at that time.

To determine if real-time classification performance is feasible with computation offloaded implementations of emotion classifiers, we measured the amount of time required to process a 90-second video recorded at 30 frames per second and subsampled to one frame per second, yielding a total of 90 frames. For each classifier, this experiment was repeated 10 times to obtain the average number of seconds required to process the subsampled video. Table 4 shows the speed of the API-based classifiers used in this study. The values shown in this table represent the amount of time necessary to send each frame to the Web service via an http post request and receive an http response with the emotion label. These frames were processed sequentially, with no overlap between http requests.

**Table 4:** Speed of the evaluated classifiers.

Classifier	Time (seconds)
Azure	28.6
AWS	90.6
Google	55.9
Sighthound	41.1

The findings indicated that the fastest classifier was Azure, processing all 90 frames in a total of 28.6 seconds. Using Azure with a fast internet connection, it may be possible to obtain semi real-time emotion classification performance, a time of 28.6 seconds corresponds to 3.14 frames per second, which is within the bounds of what could be considered real time. The slowest classifier was AWS, which processed these 90 frames in 90.6 seconds. This corresponds to a frame rate of 0.99 frames per second. In summary, real-time or semi–real-time performance is possible with Web-based emotion classifiers on fast Wi-Fi internet connections. For cellular connections or apps that require frame rates beyond three frames per second, these approaches may be insufficient.

## Discussion

### Classifier Performance

Results indicate that Google and AWS produced the highest percentage of correctly classified emotive frames, whereas Sighthound produced the highest percentage of correctly identified neutral frames. Google’s API did not provide a *neutral* label and, therefore, could not be evaluated. The best system in terms of overall classification accuracy was Sighthound by a small margin, with 60.18% (1566/2602) of the frames correctly identified. Further results indicate that none of the classifiers performed well for any category besides *happy*, which was the emotion most represented in the dataset, as shown in Table 1. In addition, there appears to be a systematic bias toward high recall and low precision for the *happy* category: those classifiers that identified most of the *happy* frames performed worse for those in the *neutral* category.

In summary, the data suggest that although a frame with a smile will be correctly identified in most cases, the ability of the evaluated classifiers to identify other expressions for children with ASD is dubious and presents an obstacle in the design of emotion-based mobile and wearable outcome measures, screening tools, and therapies.

### Analysis of Frames

**Figure 7:**
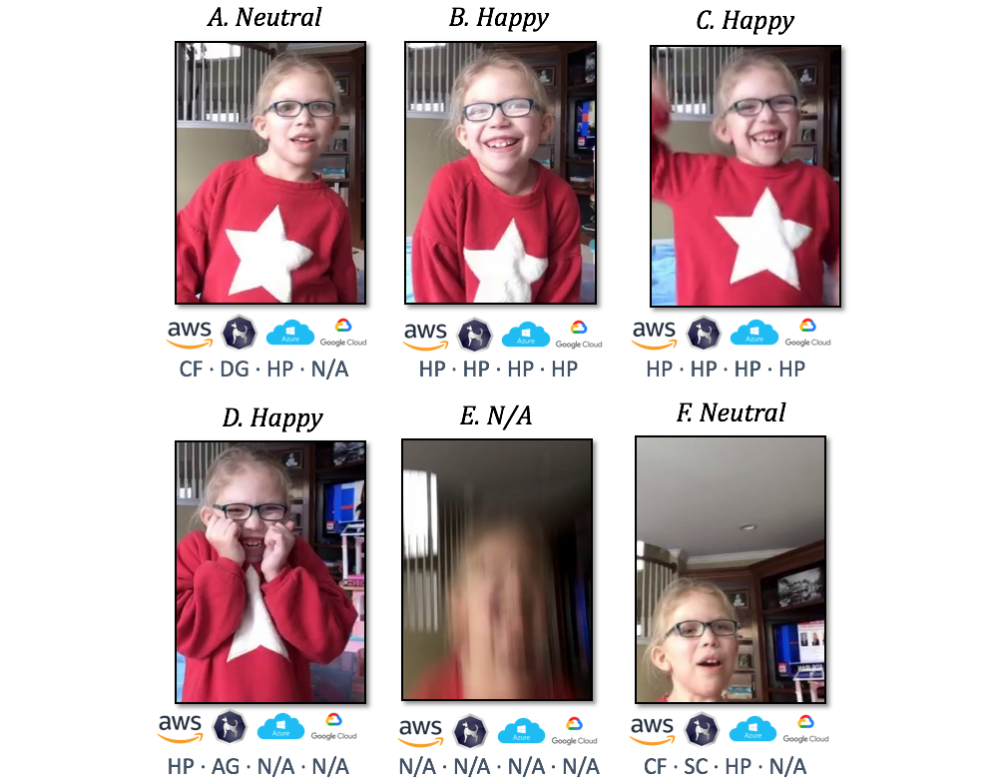
A comparison of the performance of each classifier on a set of frames highlights scenarios that may lead to discrepancies in the classifier outputs for various emotions: HP (Happy), CF (Confused), DG (Disgust), N/A (Not Applicable), AG (Angry), SC (Scared). Ground truth manual labels are shown on top, with labels derived from each classifier on the bottom.

Figure 7 shows six frames from one study participant, reproduced with permission from the child’s parents. The top of each frame lists the gold-standard annotation in which both raters agreed on a suitable label for the frame. The bottom of each frame enumerates the labels assigned from each classifier in order: Amazon Rekognition, Sighthound, Azure Cognitive Services, and Google Cloud Vision AI. It should be noted that, as before, these labels are normalized for comparison because each classifier outputs data in a particular format. For example, *N/A* from one classifier could be compared with a blank field in another, whereas some such as Google Cloud explicitly state *Not Sure*; for our purposes, all three of these scenarios were labeled as *N/A* during analysis.

Frame A shows a frame that was labeled as *neutral* by the raters, although each classifier provided a different label: *confused*, *disgusted*, *happy*, and *N/A*. This is an example of a false-positive, detecting an emotion in a neutral frame. A similar example is shown in frame F; most classifiers failed to identify the neutral label. Such false positives are particularly problematic as the *neutral* label is the most prevalent, as shown in Table 1.

In contrast, frames B and C are examples in which the labels assigned by each classifier matched the labels assigned by the manual raters; all classifiers correctly identified the *happy* label. As shown in Table 1, *happy* was the most common nonneutral emotion by a considerable margin, and most classifiers performed quite well in this category; AWS, Azure, and Sighthound all correctly identified between 78.2% (676/864) and 82.0% (709/864) of these frames, although at the expense of increased false-positives such as those shown in frames A and F. An example of a *happy* frame that was identified as such by the human raters but incorrectly by most classifiers is frame D. It is possible that the child’s hands covering part of her face contribute to this error, as the frame is otherwise quite similar to frame B. Finally, frame E is an example of a frame that was processed by the classifiers but not included in our experimental results because the human raters flagged the frame as insufficient due to motion artifacts. In this case, all four classifiers correctly determined that the frame could not be processed.

### Limitations

There are several limitations associated with this study, which will be addressed in future work. First, we analyzed only a subset of existing emotion classifiers, emphasizing those from providers of major cloud services. Future efforts will extend this evaluation to include those that are less prolific and require paid licenses. A second limitation is the use of parent-reported diagnoses, which may not always be factual. A third limitation is that although we ruled out some comorbid conditions, we did not rule out all comorbid conditions, including Attention-Deficit/Hyperactivity Disorder, which has been shown to impact emotional processing and function in children [64]. A fourth limitation stems from the lack of neurotypical children. Finally, the dataset we used included an unequal distribution of frames across emotion categories. In the future, we will investigate ways to gather equal numbers of frames, and if this distribution may be related to social deficits associated with autism, or increased prevalence of happy and neutral due to the inherent nature of gameplay. Although our results support the conclusion that the commercial emotion classifiers tested here are not yet at a level needed for use with autistic children, it remains unclear how these models will perform with a larger, more diverse, and stratified sample.

### Conclusions

In this feasibility study, we evaluated the performance of four emotion recognition classifiers on children with ASD: Google Cloud Vision, Amazon Rekognition, Microsoft Azure Emotion API, and Sighthound. The average percentage of correctly identified emotive and neutral frames for all classifiers combined was 63.60% (769/1209) and 55.63% (775/1393), respectively, varying greatly between classifiers based on how their sensitivity and specificity were tuned. The results also demonstrated that while most classifiers were able to consistently identify *happy* frames, the performance for *sad*, *disgust*, and *anger* was poor: no classifier identified more than one-third of the frames from either of these categories. We conclude that the performance of the evaluated classifiers is not yet at the level for use in mobile and/or wearable therapy solutions for autistic children, necessitating the development of larger training datasets from these populations to develop more domain-specific models.

## Abbreviations

ABA: applied behavior analysis
AI: artificial intelligence
API: application programming interface
ASD: autism spectrum disorder
ESDM: early start Denver model
SH: Sighthound
